# Prone positioning improves ventilation–perfusion matching assessed by electrical impedance tomography in patients with ARDS: a prospective physiological study

**DOI:** 10.1186/s13054-022-04021-0

**Published:** 2022-05-27

**Authors:** Yu-xian Wang, Ming Zhong, Min-hui Dong, Jie-qiong Song, Yi-jun Zheng, Wei Wu, Jia-le Tao, Ling Zhu, Xin Zheng

**Affiliations:** 1 Department of Critical Care Medicine, Zhongshan Hospital of Fudan University, Shanghai, China; 2 Shanghai Institute of Infectious Disease and Biosecurity, School of Public Health, Fudan University, Shanghai, China; 3 Shanghai Committee of Science and Technology (21MC1930400), Shanghai, China

**Keywords:** Electrical impedance tomography, Prone positioning, Acute respiratory distress syndrome, Ventilation–perfusion matching, Mechanical ventilation, Pulmonary perfusion

## Abstract

**Background:**

The physiological effects of prone ventilation in ARDS patients have been discussed for a long time but have not been fully elucidated. Electrical impedance tomography (EIT) has emerged as a tool for bedside monitoring of pulmonary ventilation and perfusion, allowing the opportunity to obtain data. This study aimed to investigate the effect of prone positioning (PP) on ventilation–perfusion matching by contrast-enhanced EIT in patients with ARDS.

**Design:**

Monocenter prospective physiologic study.

**Setting:**

University medical ICU.

**Patients:**

Ten mechanically ventilated ARDS patients who underwent PP.

**Interventions:**

We performed EIT evaluation at the initiation of PP, 3 h after PP initiation and the end of PP during the first PP session.

**Measurements and main results:**

The regional distribution of ventilation and perfusion was analyzed based on EIT images and compared to the clinical variables regarding respiratory and hemodynamic status. Prolonged prone ventilation improved oxygenation in the ARDS patients. Based on EIT measurements, the distribution of ventilation was homogenized and dorsal lung ventilation was significantly improved by PP administration, while the effect of PP on lung perfusion was relatively mild, with increased dorsal lung perfusion observed. The ventilation–perfusion matched region was found to increase and correlate with the increased PaO_2_/FiO_2_ by PP, which was attributed mainly to reduced shunt in the lung.

**Conclusions:**

Prolonged prone ventilation increased dorsal ventilation and perfusion, which resulted in improved ventilation–perfusion matching and oxygenation.

*Trial registration*: ClinicalTrials.gov, NCT04725227. Registered on 25 January 2021.

**Supplementary Information:**

The online version contains supplementary material available at 10.1186/s13054-022-04021-0.

## Introduction

Prone positioning (PP) has been used for a long time in ARDS patients who received mechanical ventilation for oxygenation improvement and lung protection purposes [1, 2] and has been shown associated with improved survival in selected ARDS patients [3]. Physiologically, PP reduces ventilator-induced lung injury (VILI) by improving lung homogeneity, promoting alveolar recruitment and reducing hyperinflation [4]. For oxygenation improvement, PP restores lung aeration and decreases shunting while preserve perfusion in dorsal lung regions without impairing gas exchange in ventral regions, consequently improving ventilation–perfusion matching in the injured lung [5–8]. However, although these physiological findings have been widely acknowledged, most of them were derived from animal models, and direct evidence has rarely been obtained in patients due to the lack of proper harmless evaluation methods over the past decades.

The emergence of electrical impedance tomography (EIT) has been promising in removing the bottleneck. As a radiation-free and noninvasive technique, EIT, which images regional impedance distribution in a cross-sectional area of the body, provides real-time visualization of lung ventilation at the bedside [9]. In addition, contrast-enhanced EIT allows concurrent estimation of regional pulmonary perfusion based on the kinetics of a bolus of hypertonic saline contrast injected through a central venous line [10, 11]. With these characteristics, EIT is no doubt an ideal tool for dynamic evaluation of pulmonary physiological changes, especially for regional ventilation–perfusion matching in ARDS patients, which remains poorly illustrated. Recently, studies involving EIT for lung ventilation assessment have revealed notable effects of PP on ventilatory mechanics in ARDS patients, including regional gas redistribution, improved alveolar recruitment, and lung homogeneity [7, 8, 12, 13], even though few study has set to demonstrate the influence on pulmonary ventilation–perfusion matching during PP in ARDS patients.

In this study, we aimed to evaluate the impact of PP on ventilation and pulmonary perfusion in patients with moderate-to-severe ARDS using contrast-enhanced EIT, trying to unveil the underlying physiological alterations beyond the improved oxygenation to some extent.

## Materials and methods

### Patients

This prospective study was conducted in the intensive care unit (ICU) of Zhongshan Hospital, Fudan University, and was approved by the Institutional Ethics Committee of the hospital (NO.B2019-230R). All patients were recruited upon obtaining written informed consent. The inclusion criteria included moderate-to-severe ARDS patient (defined as PaO_2_/FiO_2_ < 150 mmHg with positive end-expiratory pressure (PEEP) ≥ 5 cmH_2_O, and FiO_2_ ≥ 0.6 according to the Berlin definition [14]) who underwent mechanical ventilation and PP upon clinical decision-making within the first PP session. The exclusion criteria included age < 18 years, severe hemodynamic instability, pregnancy, PP length < 12 h, contraindications to EIT administration, or the inability of EIT belt placement. This study is registered at clinicaltrials.gov (NCT04725227).

### Study protocol

The following information on the baseline characteristics of the patients was collected at enrollment: sex, height, body mass index (BMI), acute physiology and chronic health evaluation II (APACHE II) score at ICU admission, etiology of ARDS, and the arterial partial oxygen pressure to fractional concentration of inspired oxygen (PaO_2_/FiO_2_) ratio. Before PP upon clinical decision-making, patients were initially transported to the CT scan facility in the supine position.

EIT assessment was administered in the supine position (T_0_), 3 h after PP initiation (T_1_), and at the end of PP (T_2_) within the first PP session. Arterial blood gas (ABG) analysis results, end-tidal expiratory carbon dioxide pressure, ventilator parameters, and hemodynamic parameters, including heart rate (HR), central venous pressure (CVP), and mean arterial pressure (MAP), were also recorded at T_0_, T_1_, and T_2_. Patients were deeply sedated, paralyzed, and mechanically ventilated in synchronized intermittent mandatory ventilation (SIMV) mode. Ventilator settings were standardized for all patients during all study measures as follows: tidal volume (Vt) 6–8 mL/kg of predicted body weight, respiratory rate to maintain pH between 7.35 and 7.45, and PEEP based on EIT[15, 16] in the supine position and unchanged during the PP period.

### EIT data

The EIT belt containing 16 electrodes was placed around the chest wall at the fourth or fifth intercostal space and connected to the EIT monitor (PulmoVista 500; Dräger Medical GmbH, Lübeck, Germany). Technical details of EIT have been previously described [11]. The EIT signals were recorded at frame rate of 50 Hz. After a baseline recording of EIT data for 5 min, we performed an end-inspiratory breath hold lasting 20 s. Two seconds after the start of the occlusion, a bolus of 10 ml of 5% NaCl solution was manually injected via the central venous catheter. The bolus of saline solution, injected in less than 2 s, passes through the pulmonary circulation producing an impedance dilution curve that follows typical first-pass kinetics [17]. EIT ventilation maps (Additional file 1: Fig. S1A) were obtained from offline analysis of tracings by averaging values over five consecutive respiratory cycles. Ventral and dorsal regions were defined as the upper and lower parts of an axis from the sternum to the vertebrae, respectively. From the analysis of ventilation maps, we took the following measurements [18]:

1. the pixel-level ventilation is measured as impedance change between expiration and inspiration. Pixels were then classified as nonventilated if pixel ventilation was ≤ 10% of the highest pixel-level value measured in that patient.
2. Tidal image region-global (%) = the relative pixel-level ventilation / the relative pixel-level detected lung size [19].
3. The percentage of ventilated pixels in the respective region (region of interest: ROI 1, ROI 2, ROI 3and ROI 4) (Additional file 1: Fig. S1D).
4. Ventral/ Dorsal of tidal image region(%) = Tidal image region-global × the ventral/ dorsal fraction of ventilation distribution
5. The Global Inhomogeneity (GI) index [20]

EIT perfusion maps (Additional file 1: Fig. S1B) were derived from offline analysis of the time-impedance curve obtained during the first pass of the saline solution during occlusion after removing the cardiac region from the images. From the analysis of perfusion maps, the following measurements were taken [18]:

1. Regarding relative pixel-level perfusion, after preprocessing, the steepest (maximal) slope of the temporal EIT signal deflection during saline bolus injection in each pixel was normalized to the overall detected signal, yielding the relative pixel perfusion. Pixels were classified as nonperfused if pixel perfusion was ≤ 10% of the highest pixel-level value measured in that patient.
2. Blood flow region-global (%) = the relative pixel-level perfusion / the relative pixel-level detected lung size.
3. The percentage of perfusion in each region (ROI 1, ROI 2, ROI 3 and ROI 4) (Additional file 1: Fig. S1D).
4. Ventral/ dorsal of blood flow region (%) = Blood flow region-global (%) × the ventral/ dorsal fraction of perfusion distribution.
5. The GI index [20].

By integrating the pixel-level data on ventilation and perfusion (Additional file 1: Fig. S1C), we calculated the following:

1. Dead Space-EIT %, corresponding to the ventilated but nonperfused pixels divided by the total number of pixels ventilated and/or perfused,
2. Shunt-EIT %, corresponding to the perfused but nonventilated pixels divided by the total number of pixels classified as ventilated and/or perfused,
3. Matched Region %, corresponding to the pixels that are both ventilated and perfused divided by the total number of pixels ventilated and/or perfused.

**Fig. 1:**
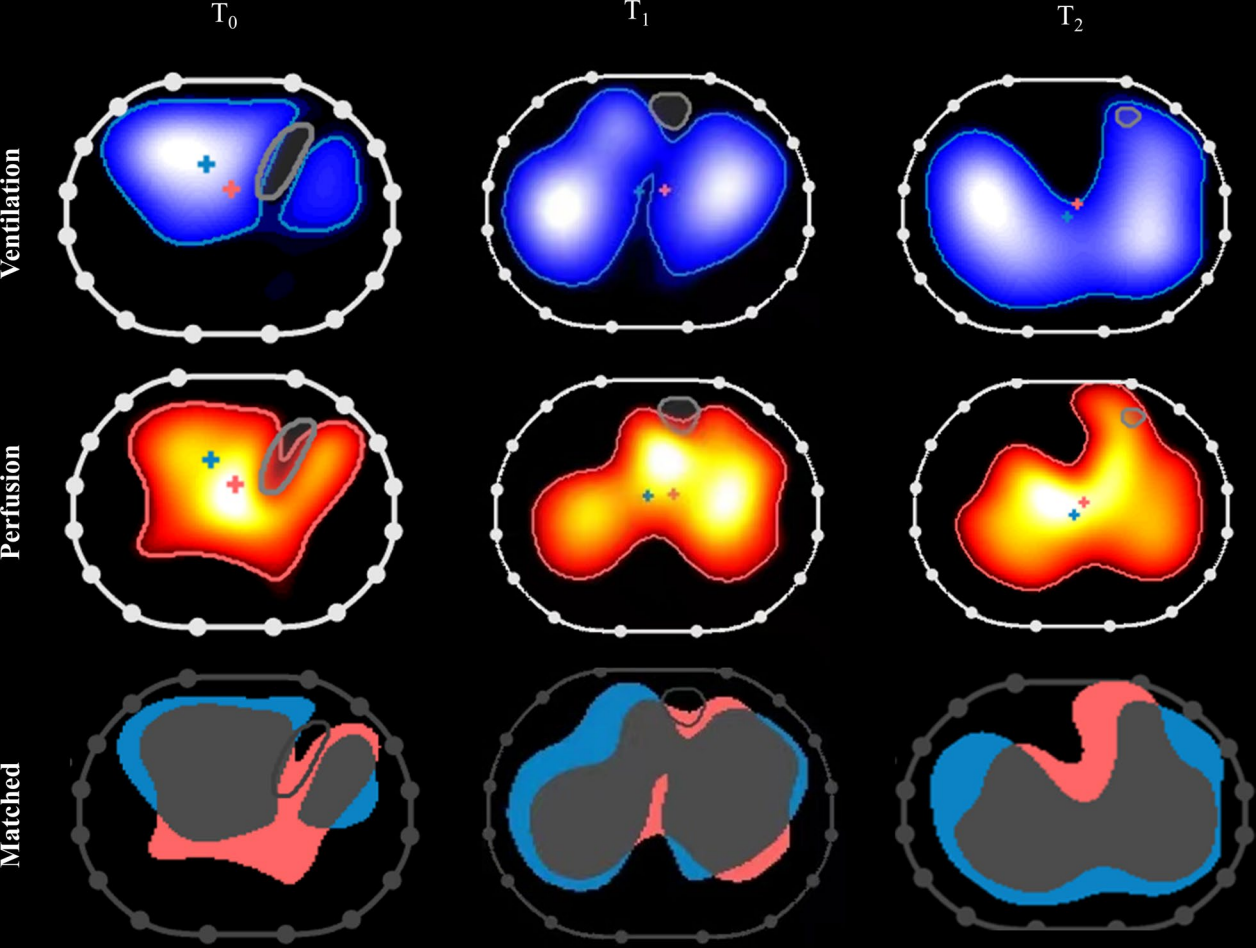
Effects of prone position on ventilation–perfusion matching in a representative study patient. Left: performed in the supine (T_0_), Middle: 3 h after PP initiation (T_1_), Right: at the end of PP (T_2_). During prone ventilation, pulmonary blood flow is mostly unmodified at the early stage and then gradually changes over time. Long prone ventilation increases dorsal ventilation and perfusion, which results in improved ventilation–perfusion matching

Figure 1 shows ventilation and perfusion matching images of a representative patient at T_0_, T_1,_ and T_2_.

### Study endpoints

The aims of the study were as follows:

1. To explore the effect of PPV on ventilation–perfusion matching. The primary endpoints were Dead Space-EIT %, Shunt-EIT %, and Matched Region %.
2. To explore the regional distribution of ventilation and perfusion during the prone position in patients with moderate-to-severe ARDS using contrast-enhanced EIT.

### Statistical analysis

The sample size was similar to previous physiologic studies [21, 22]. Statistical analysis was performed via SPSS 26.0 (SPSS Inc. Chicago, IL) and Prism 8 (GraphPad Software, San Diego, CA, USA). All continuous variables were subjected to Shapiro–Wilk test for normality. Data are presented as the mean ± SD if normality was met or otherwise as the median and interquartile range. To test the effect of the time points on the variables, Mauchly’s test was performed for sphericity, and repeated measures ANOVA was applied with post hoc Bonferroni’s multiple comparisons. When the violation of sphericity occurred (i.e., Mauchly’s test *p* value < 0.05), the Greenhouse–Geisser method was used for correction. Correlation between continuous variables was assessed by the Pearson regression coefficient. All statistical tests were two-tailed, and *p* < 0.05 was considered statistically significant.

## Results

### Patient characteristics

A total of 10 ARDS patients who underwent mechanical ventilation in the ICU of Zhongshan Hospital, Fudan University, from February 2021 to July 2021 were enrolled (Table 1). The patients consisted of 9 male and 1 female, aged 66 ± 15 years with an average body mass index (BMI) of 24 kg/m^2^. The average APACHE II score at ICU admission was 14 ± 9. Three patients (30%) had moderate ARDS, and 7 patients (70%) had severe ARDS. All patients underwent continuous PP with an average duration of 15.45 ± 2.5 h. More detailed clinical characteristics of the study population are presented in Additional file 1: Table S1 and Table S2.

**Table 1:** Main characteristics of the patients

Patient NO	Sex (M/F)	Height (cm)	BMI (kg/m^2^)	Comorbidities	APACHE II score at ICU admission	PaO_2_/FiO_2_ at baseline (mm Hg)	PEEP (cmH_2_O)	MV duration before enrollment (d)	Duration of prone positioning (h)	ARDS etiology
1	M	178	25	Hypertension	33	83	10	1	12	Pneumonia
2	M	173	26	None	11	123	8	3	17	Pneumonia
3	F	162	35	Hypertension; immunodeficiency	9	51	14	1	18.5	Septic shock
4	M	165	24	Hypertension; diabetes	19	81	11	4	16	Postoperative respiratory failure
5	M	164	23	Hypertension; atrial fibrillation	15	97	8	4	18.5	Pneumonia
6	M	182	21	None	2	97	8	5	16	Pneumonia
7	M	170	21	None	7	140	5	2	15.5	Postoperative respiratory failure
8	M	168	26	Hypertension; asthma	11	96	5	7	12	Pneumonia
9	M	170	19	Hypertension	23	64	12	2	16.5	Pneumonia
10	M	163	23	Hypertension	8	122	12	3	12.5	Septic shock
Summary	9M/1F	169.5 ± 6.6	24 ± 4		14 ± 9	95.4 ± 27	9.3 ± 3.02	3.2 ± 1.8	15.45 ± 2.5	

M, male; F, female; BMI, Body Mass Index; APACHE-II, Acute Physiology and Chronic Health Evaluation II; ICU, intensive care unit; PaO_2_/FiO_2_, arterial partial pressure of O_2_/inspired fraction of O_2_ ratio; PEEP, positive end-expiratory pressure; MV, mechanical ventilation; ARDS, acute respiratory distress syndrome

### PP improves oxygenation and hemodynamic stability during mechanical ventilation

Clinical parameters regarding respiratory and hemodynamic status at T_0_, T_1_, and T_2_ were assessed and compared (Table 2). Notably, with PP administration, the respiratory system compliance (Crs) (27.70 vs. 30.03 vs. 33.52 ml/cmH_2_O, *p* < 0.01) and PaO_2_/FiO_2_ (95.40 vs. 161.90 vs. 221.40 mmHg, *p* < 0.0001) of the patients significantly increased. No significant difference was identified in minute ventilation (MV) (*p* = 0.536), PaCO_2_ (*p* = 0.257), end-tidal expired carbon dioxide pressure (ETCO_2_) (*p* = 0.348) or dead space percentage estimated by ABG (Dead space-ABG) (*p* = 0.740) among the 3 time points.

**Table 2:** Effects of prone position ventilation on respiratory, gas exchange, and hemodynamics

	T_0_	T_1_	T_2_	Trend*-P* value	Mauchly’s test of sphericity *P* value
MV (l/min)	7.47 ± 1.09	7.29 ± 0.92	7.62 ± 1.32	0.536	< 0.001
Crs (ml/cmH_2_O)	27.70 ± 11.65	30.03 ± 11.36	33.52 ± 12.87^a^	< 0.01	0.061
Set FiO_2_	0.70 [0.60–0.89]	0.60 [0.59–0.69]	0.60 [0.40–0.65]	< 0.05	0.441
PaO_2_ (mmHg)	66.98 ± 12.15	100.44 ± 20.54^a^	125.35 ± 34.85^a^	< 0.0001	0.119
PaO_2_/FiO_2_ (mmHg)	95.40 ± 27.45	161.90 ± 44.68^a^	221.40 ± 54.64^a, b^	< 0.0001	0.957
PaCO_2_ (mmHg)	50.55 [41.50–63.35]	56.45 [46.33–69.93]	49.90 [42.88–68.58]	0.257	0.046
ETCO_2_ (mmHg)	36.00 [34.00–48.75]	41.00 [35.00–50.00]	37.50 [33.00–43.25]	0.348	0.010
Dead space-ABG	26.84 [17.66–34.42]	24.56 [21.64–33.43]	20.02 [17.61–37.17]	0.740	0.003
HR (bpm)	113.30 ± 19.81	108.00 ± 20.56	106.90 ± 24.53	0.387	0.198
SBP (mmHg)	116.20 ± 17.50	120.50 ± 17.80	121.60 ± 19.00	0.763	0.070
MAP (mmHg)	76.93 ± 15.97	84.83 ± 13.77	84.60 ± 17.33	0.360	0.092
CVP (mmHg)	10.00 [8.00–14.50]	14.00 [10.50–15.00]	13.00 [11.00–15.00]	0.123	0.253
Norepinephrine (µg/kg/min)	0.17 [0–0.175]	0.08 [0–0.175]	0.02 [0–0.088]^a^	< 0.05	0.377

MV, minute ventilation; PaO_2_/FiO_2_, arterial partial pressure of O_2_/inspired fraction of O_2_ ratio; PaCO_2_, arterial partial pressure of CO_2_; ETCO_2_, end-tidal expired carbon dioxide pressure; ABG, arterial blood gas; HR, heart rate; SBP, systolic arterial blood pressure; MAP, mean arterial pressure; CVP, central venous pressure. Respiratory system static compliance (Crs) = Vt/(P_plat_ − PEEP_tot_) from the analysis of ventilation tracings during occlusions; Vt, tidal volume; P_plat_, plateau pressure; PEEP_tot_, total positive end-expiratory pressure. T_0_: in the supine position; T_1_: 3 h after PP initiation; T_2_: at the end of PP

p value by one-way analysis of variance (ANOVA) for repeated measures

^a^vs. T_0_, *p* < 0.05

^b^vs. T_1_, *p* < 0.05

For hemodynamic parameters, a decrease in heart rate (HR) and increases of systolic blood pressure (SBP), MAP and CVP were observed but with no significant difference reached. However, the dose of norepinephrine significantly decreased after the initiation of PP (*p* < 0.05), indicating the improvement in hemodynamic stability in the patients (Additional file 1: Fig. S2 shows the change in norepinephrine dose during the prone position for each patient).

### PP alters the regional distribution of lung ventilation and perfusion

**Fig. 2:**
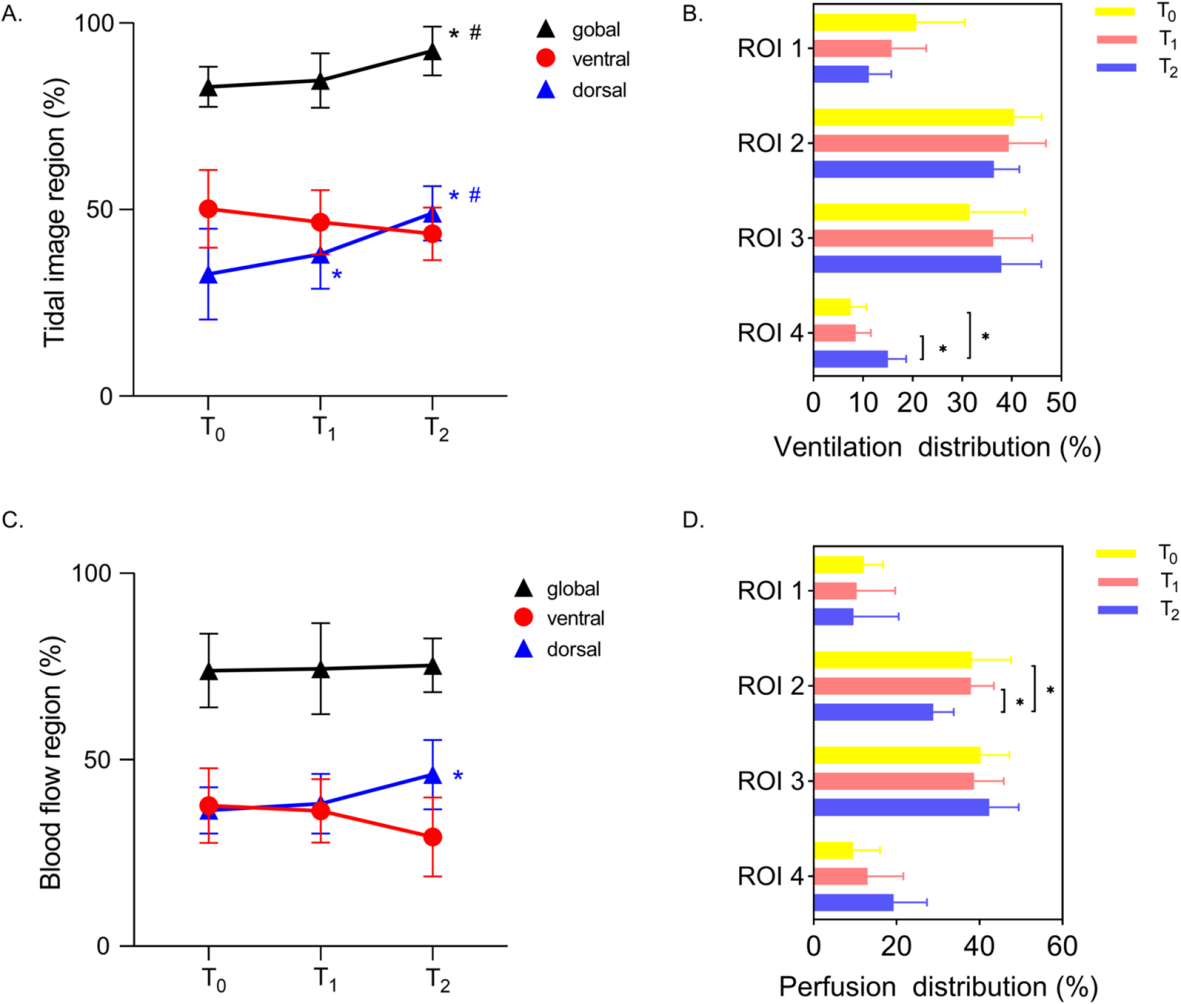
Evolution of Tidal image region (%) (**A**), Blood flow region (%) (**C**), ventilation (**B**) or perfusion distribution (%) (**D**) in the horizontal regions of interest (ROIs) at T_0_, T_1_, and T_2_. *vs. T_0_, *p* < 0.05. ^#^vs. T_1_, *p* < 0.05

EIT-based measurements regarding lung ventilation and perfusion at the indicated time points are presented in Table 3. Tidal image region-global% significantly increased with PP administration (82.9% vs. 85.6% vs. 92.5%, *p* < 0.0005). The increments took place mainly in the dorsal region (32.7% vs. 38.0% vs. 49.0%, *p* < 0.005), while no significant decrease in the ventral region was observed (Fig. 2A). Similarly, the tendency was also presented on the scale of the 4 ROIs (Fig. 2B). Moreover, the GI index of ventilation progressively decreased from T_0_ to T_2_ (*p* < 0.01, Table 3).

**Table 3:** Physiologic variables at the three different time points selected for the analysis of electrical impedance tomography data

Variables	T_0_	T_1_	T_2_	Trend*-p* value	Mauchly’s test of sphericity *p* value
Tidal image region-global (%)	82.90 ± 5.40	84.60 ± 7.31	92.50 ± 6.52^a, b^	< 0.0005	0.709
Ventral of tidal image region (%)	50.20 ± 10.44	46.60 ± 8.61	43.50 ± 7.03	0.140	0.030
Dorsal of tidal image region (%)	32.70 ± 12.14	38.00 ± 9.24^a^	49.00 ± 7.32^a, b^	< 0.005	0.001
ROI 1 of ventilation distribution (%)	20.8 ± 9.7	15.8 ± 7.0	11.2 ± 4.5	< 0.05	0.099
ROI 2 of ventilation distribution (%)	39.00 [36.50–43.00]	37.00 [35.25–41.25]	37.00 [31.00–42.00]	0.315	0.030
ROI 3 of ventilation distribution (%)	31.50 ± 11.20	36.30 ± 7.85	37.90 ± 8.06	0.243	0.013
ROI 4 of ventilation distribution (%)	7.50 ± 3.24	8.50 ± 3.10	15.00 ± 3.74^a, b^	< 0.001	0.833
GI index-ventilation (%)	0.53 ± 0.06	0.52 ± 0.07	0.47 ± 0.05^a^	< 0.01	0.266
Blood flow region-global (%)	73.9 ± 9.9	74.4 ± 12.2	75.3 ± 7.2	0.817	0.022
Ventral of blood flow region (%)	37.70 ± 10.02	36.30 ± 8.52	29.30 ± 10.59	< 0.05	0.102
Dorsal of blood flow region (%)	36.42 ± 6.20	38.20 ± 7.98	46.00 ± 9.29^a^	< 0.01	0.623
ROI 1 of perfusion distribution (%)	12.10 ± 4.66	10.40 ± 9.28	9.60 ± 10.89	0.742	0.554
ROI 2 of perfusion distribution (%)	38.20 ± 9.35	37.90 ± 5.59	28.80 ± 4.96^a, b^	< 0.01	0.268
ROI 3 of perfusion distribution (%)	40.20 ± 7.00	38.70 ± 7.13	42.30 ± 7.15	0.076	0.280
ROI 4 of perfusion distribution (%)	9.50 ± 6.49	13.00 ± 8.65	19.30 ± 8.03	< 0.05	0.148
GI index-perfusion (%)	0.60 ± 0.12	0.59 ± 0.08	0.59 ± 0.10	0.993	0.088
Matched region (%)	52.50 ± 6.65	61.10 ± 7.39^a^	67.40 ± 7.09^a, b^	< 0.0005	0.468
Dead space-EIT (%)	18.00 ± 4.69	18.10 ± 5.02	14.30 ± 5.89	0.099	0.154
Shunt-EIT (%)	29.50 ± 7.56	20.80 ± 6.71^a^	18.30 ± 6.06^a^	< 0.005	0.023

ROI, Region of Interest; GI, Global Inhomogeneity. T_0_: in the supine position; T_1_: 3 h after PP initiation; T_2_: at the end of PP

p value by one-way analysis of variance (ANOVA) for repeated measures

^a^vs. T_0_, *p* < 0.05

^b^vs. T_1_, *p* < 0.05

For lung perfusion, no obvious change was found in the global or ventral regional distribution of blood flow determined by EIT, but intriguingly, perfusion in the dorsal region was shown to be significantly increased with PP duration (36.20% vs. 38.20% vs. 46.00%, *p* < 0.05) (Fig. 2C). Similarly, lung perfusion slightly decreased in ventral ROIs 1 and 2 but slightly increased in dorsal ROIs 3 and 4, although no statistical significance was reached (Fig. 2D). In addition, the GI index of perfusion remained constant during the PP period (Table 3). These results indicated that the overall effect of PP on lung perfusion distribution was relatively mild, but PP could still remarkably increase perfusion in the dorsal region of the lung.

### PP improves ventilation–perfusion matching

**Fig. 3:**
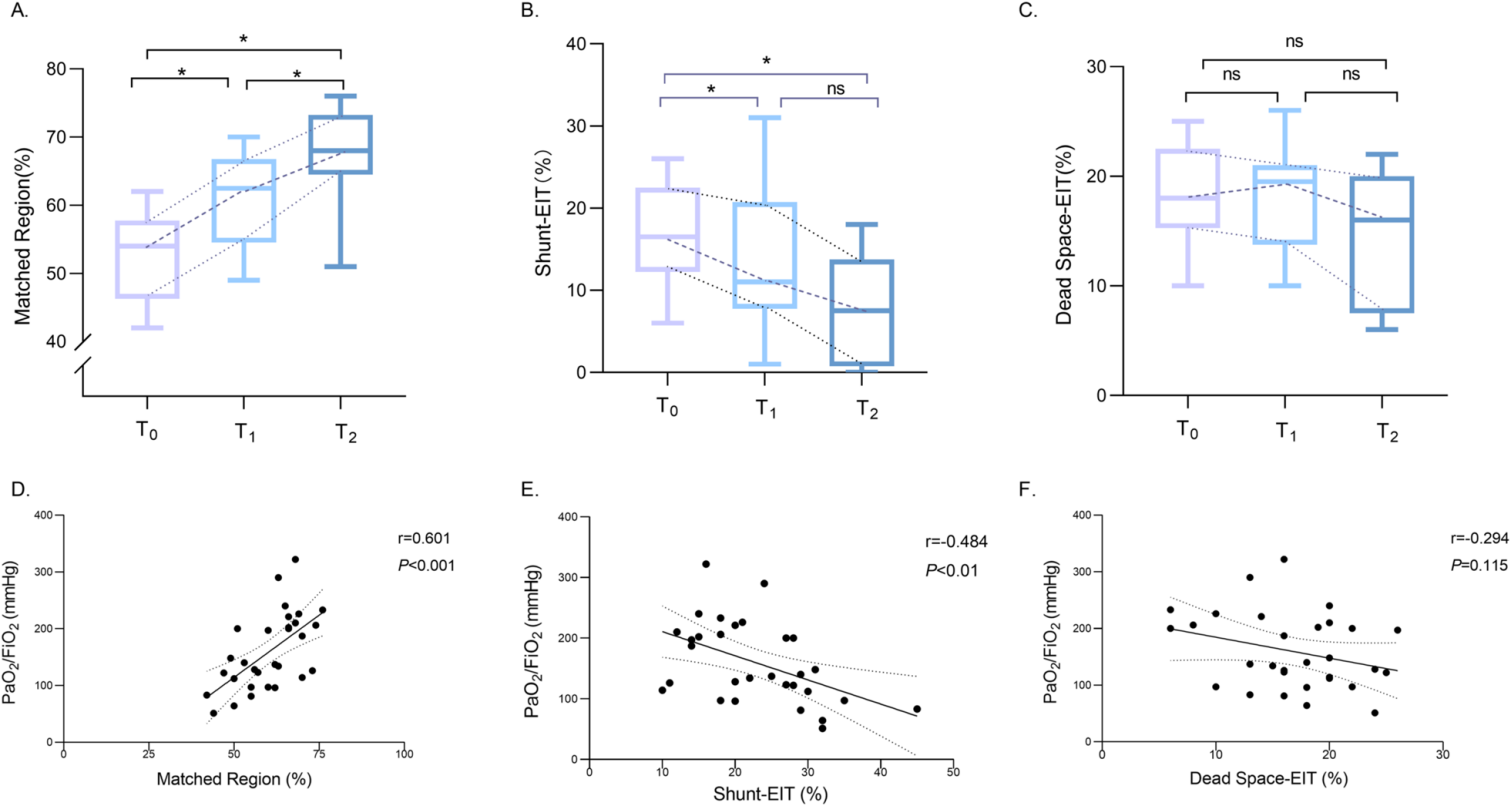
Comparisons of Matched Region (%) (**A**), Shunt-EIT (%) (**B**), and Dead Space-EIT (%) (**C**) at T_0_, T_1_, and T_2_. *p < 0.05. PaO_2_/FiO_2_ was significantly correlated with Matched Region (%) (**D**) and Shunt-EIT (%) (**E**), but not with Dead Space-EIT(%) (**F**)

Based on EIT measurements, Matched Region% significantly increased with PP duration (52.50% vs. 61.10% vs. 67.40%, *p* < 0.0005) (Fig. 3A). Intriguingly, Shunt-EIT % significantly decreased with PP duration (29.50% vs. 20.80% vs. 18.30%, *p* < 0.005, Fig. 3B), especially between T_0_ and T_1_. Dead Space-EIT % remained almost the same during PP administration (Fig. 3C) (Additional file 1: Fig. S3 shows the changes of Matched Region%, Shunt-EIT % and Dead Space-EIT % in different time points, for individual). As shown in Fig. 3D, E, F, PaO_2_/FiO_2_ presented a positive correlation with Matched Region % (rho = 0.601, *p* < 0.0005) and negative correlation with the level of shunt estimated by EIT (rho = − 0.484, *p* < 0.01), while there was no significant correlation with Dead Space-EIT %. Moreover, in our study, most of the patients’ lungs had good recruitability by from CT scan after three PP sessions (Additional file 1: Figure S4). Two patients lacked CT scans due to their clinical severity and abandonment of treatment.

## Discussion

In use of EIT, this study has provided some proof-of-concept evidence regarding the ventilation–perfusion matching effect of PP during mechanical ventilation in classical ARDS patients. The main findings can be summarized as follows. (1) The increased ventilation was distributed mainly to the dorsal region, while ventral ventilation was hardly changed, suggesting that the aeration increment in dorsal lung regions was not offset by the loss of aeration in ventral regions. Changes in ventilation were observed early in the prone position. (2) The redistribution effect of PP on lung perfusion was relatively mild compared to ventilation, with the dorsal lung perfusion increased and the trend of ventral perfusion decreased, while global perfusion remained constant. Changes in blood flow were observed during prolonged prone positions. (3) The ventilation–perfusion matched region was increased and correlated with the increased PaO_2_/FiO_2_ by PP, which was specifically attributed to reduced functional shunt and increased dorsal ventilation and perfusion matching by the procedure.

In classic ARDS, with the lung less compliant with the four- to fivefold increase in its mass, the superimposed pressure increases the pleural pressure gradient substantially with consequent severe compression atelectasis in gravitationally dependent lung regions and regional overdistension in the nondependent lung [23, 24]. Therefore, in the prone position, these alveoli may open up as the superimposed pressure of the dorsal regions is released [25]. In the present study, the patients exhibited a shift in the tidal volume distribution from the ventral to dorsal ROIs in the early stages of PP, with the GI index reduced. Previous studies investigated whether the inflation and aeration of pulmonary units was far more homogeneous at pronation than supination, which was interpreted as the lung-distending force (i.e., the gravitational gradient of pleural pressure, transpulmonary pressure, or lung stress) was more homogeneously distributed [25–27]. The primary reason was considered to be improved shape matching between the chest wall and the lung, which is the so-called ‘sponge-lung’ phenomenon [25, 28]. From a physiologic point of view, PP might be a more effective and safer approach to revert dorsal loss of ventilation.

The human lung, as with other mammalian lungs, is structurally optimized to function in the prone posture, in terms of both gross lung shape and vascular architecture. Theoretically, the gravitational distribution of pulmonary blood flow is only minimally altered by pronation [29, 30], which is consistent with our findings. Interestingly, we discovered that during prone ventilation, pulmonary blood flow remains unmodified at the early stage and then gradually increases in dorsal regions over time in classic ARDS. The increased perfusion in the dorsal region and roughly maintained perfusion in the ventral region could be attributed to less heart superimposed pressure, less airspace compression of vessels (with more end-expiratory volume), and the reduction in hypoxic vasoconstriction in the dorsal region [5]. Perier et al. recently reported improvements in lung aeration and unmodified dorsal pulmonary perfusion after three hours of PP in COVID-19-associated ARDS [31]. In contrast, a recent case study found that both ventilation and perfusion changed one hour after a patient with acute respiratory failure secondary to SARS-CoV-2019 was turned from the supine to the prone position [32]. Therefore, it is meaningful and essential to capture changes in regional ventilation and perfusion by EIT in critically ill individuals.

In theory, the matching between lung ventilation and perfusion is fundamental to effective gas exchange, and both shunt and dead space are the determinants of ventilation–perfusion matching. Our data suggested that Matched Region% improved before the end of the prone position with the significant decrease in Shunt-EIT%, correlated with PaO_2_/FiO_2_, which was in accordance with previous studies [27, 33]. Moreover, a recent clinical study reported that after pronation for 30 min, ventral lung regions are characterized by a decreased fraction of ventilated nonperfused units and a reduced dead space/shunt ratio in patients with COVID-19-associated ARDS[34]. In the supine position, gravity and lung structure cause regional ventilation and perfusion to diverge, and this posture is thus suboptimal in disease states with pathological ventilation–perfusion mismatching, such as ‘classic’ ARDS and COVID-19-associated ARDS [35]. In our study, six patients could be categorized to the pulmonary cause (ARDS_p_) group, and four patients to the extrapulmonary cause (ARDS_exp_). In terms of the trend of change, the effect of prone position was more pronounced early in the ARDS_p_ group compared to the ARDS _exp_ group (Additional file 1: Fig. S5). However, prolonged prone ventilation finally both increases dorsal ventilation and perfusion in the lung in two groups, which results in improved ventilation–perfusion matching. Further studies are needed to validate the effect of PP at different stages of time and for different ARDS types.

Although this study has provided notable evidence regarding the physiological effects of prone ventilation in classic ARDS patients, its limitation should be noted. First, this study was carried out within a small number of patients in a single center, which reduces the generalizability of the results. Second, EIT measures only a portion of the lung, projecting the three-dimensional distribution of ventilation/perfusion to the two-dimensional image, which is not enough for assessment of the entire spectrum of ventilation–perfusion matching in the lung [36]. Third, the EIT assessment was performed only at the 3 indicated time points during PP instead of throughout the process, so it failed to fully uncover the longitudinal effect of PP. The present study also missed the changes in ventilation and perfusion after resupination. Forth, the cardiac output of the patients was not measured. The potential effect of PP on CO might influence oxygenation and V-Q matching.

## Conclusions

The bedside EIT evaluation supports the idea that prolonged prone ventilation increases dorsal ventilation and perfusion in the lung, which results in improved ventilation–perfusion matching and, consequently, oxygenation.

## Supplementary Information

**Additional file 1: Table S1.** Baseline characteristics of the patients. **Table S2.** Cardiopulmonary characteristics of the patients. **Figure S1.** Ventilation and perfusion measured by EIT in a representative patient. **A**. Representative image of the ventilation (blue-color map) distribution. **B**. Representative image of the perfusion (red-color map) distribution. **C**. Representative map obtained by integrating ventilation and perfusion maps: The gray area indicates matched units which are both ventilated and perfused, while red area indicated only perfused units and blue area only ventilated units. **D**. Representative map with the percentage of ventilation (blue numbers) and perfusion (red numbers) distribution in the four horizontal regions of interest (ROIs). This choice allowed us to obtain more superimposable regions of interest. **Figure S2.** The change in norepinephrine dose during the prone position for each patient. **Figure S3.** **A**. The change in Matched Region (%) during the prone position for each patient. **B**. The change in Shunt-EIT (%) during the prone position for each patient. **C**. The change in Dead Space (%) during the prone position for each patient. **Figure S4.** Representative chest CT images obtained before prone position and after three prone position sessions. **Figure S5.** Evolution of tidal image region (%), blood flow region (%) at T_0_, T_1_, and T_2_ in the ARDS_p_ and ARDS_exp_ groups. Six patients could be categorized to the pulmonary cause (ARDS_p_) group, and four patients to the extrapulmonary cause (ARDS_exp_). In terms of the trend of change, the effect of prone position was more pronounced early in the ARDS_p_ group compared to the ARDS_exp_ group. However, prolonged prone ventilation finally both increases dorsal ventilation and perfusion in the lung in two groups, which results in improved ventilation–perfusion matching.

## Data Availability

The datasets used and/or analyzed during the current study are available from the corresponding author on reasonable request.

## Abbreviations

ARDS: Acute respiratory distress syndrome
EIT: Electrical impedance tomography
PP: Prone positioning
PEEP: Positive end expiratory pressure
Vt: Tidal volume
RR: Respiratory rate
FiO_2_: Fraction of inspired oxygen
V/Q: Ventilation/perfusion
GI: Global Inhomogeneity
ROI: Region of Interest
