# Supplementary material for: Prone positioning improves ventilation–perfusion matching assessed by electrical impedance tomography in patients with ARDS: a prospective physiological study
Source: Crit Care. 2022 May 27;26:154. doi: 10.1186/s13054-022-04021-0 (PMC9137443; doi:10.1186/s13054-022-04021-0)
Supplement: Supplementary file 1 — Additional file 1: Table S1. Baseline characteristics of the patients. Table S2. Cardiopulmonary characteristics of the patients. Figure S1. Ventilation and perfusion measured by EIT in a representative patient. A. Representative image of the ventilation (blue-color map) distribution. B. Representative image of the perfusion (red-color map) distribution. C. Representative map obtained by integrating ventilation and perfusion maps: The gray area indicates matched units which are both ventilated and perfused, while red area indicated only perfused units and blue area only ventilated units. D. Representative map with the percentage of ventilation (blue numbers) and perfusion (red numbers) distribution in the four horizontal regions of interest (ROIs). This choice allowed us to obtain more superimposable regions of interest. Figure S2. The change in norepinephrine dose during the prone position for each patient. Figure S3. A. The change in Matched Region (%) during the prone position for each patient. B. The change in Shunt-EIT (%) during the prone position for each patient. C. The change in Dead Space (%) during the prone position for each patient. Figure S4. Representative chest CT images obtained before prone position and after three prone position sessions. Figure S5. Evolution of tidal image region (%), blood flow region (%) at T0, T1, and T2 in the ARDSp and ARDSexp groups. Six patients could be categorized to the pulmonary cause (ARDSp) group, and four patients to the extrapulmonary cause (ARDSexp). In terms of the trend of change, the effect of prone position was more pronounced early in the ARDSp group compared to the ARDSexp group. However, prolonged prone ventilation finally both increases dorsal ventilation and perfusion in the lung in two groups, which results in improved ventilation–perfusion matching. [file 13054_2022_4021_MOESM1_ESM.docx]

**Prone positioning improves ventilation-perfusion matching assessed by electrical impedance tomography in patients with ARDS: a prospective physiological study**

Yu-xian Wang^#1^, BS; Ming Zhong^#*1,2,3,^ MD; Min-hui Dong^#1^, MD; Jie-qiong Song^1^, MD; Yi-jun Zheng^1^, MS; Wei Wu^1^, MS; Jia-le Tao^1^, BS; Ling Zhu^1^, BS; Xin Zheng^1^, AD

Online supplement

# Additional Table

Table S1 Baseline characteristics of the patients

| Patient NO. | Crs (ml/cmH_2_O) | Set FiO_2_ | PaO_2_ (mmHg) | Dead space-ABG | HR (bpm) | SBP (mmHg) | MAP (mmHg) | CVP (mmHg) | Norepinephrine (µg/kg/min) |
| --- | --- | --- | --- | --- | --- | --- | --- | --- | --- |
| 1 | 51.40 | 0.85 | 71.20 | 41.94 | 121.00 | 104.00 | 56.67 | 8.00 | 0.17 |
| 2 | 26.50 | 0.70 | 86.30 | 24.33 | 135.00 | 106.00 | 80.67 | 8.00 | 0.30 |
| 3 | 30.00 | 1.00 | 51.60 | 28.57 | 80.00 | 131.00 | 91.00 |  | 0.00 |
| 4 | 27.60 | 0.70 | 56.90 | 25.11 | 90.00 | 92.00 | 63.33 | 10.00 | 0.00 |
| 5 | 23.00 | 0.60 | 60.40 | 31.03 | 140.00 | 105.00 | 74.33 | 11.00 | 0.17 |
| 6 | 22.00 | 0.70 | 68.30 | 21.05 | 130.00 | 101.00 | 69.67 | 16.00 | 0.19 |
| 7 | 33.00 | 0.60 | 84.60 | 5.26 | 100.00 | 117.00 | 67.00 | 13.00 | 0.17 |
| 8 | 14.50 | 0.60 | 53.00 | 7.50 | 109.00 | 125.00 | 69.67 | 16.00 | 0.07 |
| 9 | 38.20 | 1.00 | 64.10 | 34.36 | 105.00 | 134.00 | 85.33 | 8.00 | 0.17 |
| 10 | 10.80 | 0.60 | 73.40 | 34.58 | 123.00 | 147.00 | 111.67 | 9.00 | 0.00 |

MV, minute ventilation; PaO_2_/FiO_2_, arterial partial pressure of O_2_/inspired fraction of O_2_ ratio; PaCO_2_, arterial partial pressure of CO_2_; ABG, arterial blood gas; HR, heart rate; SBP, systolic arterial blood pressure; MAP, mean arterial pressure; CVP, central venous pressure. Respiratory system static compliance (Crs) = Vt/ (P _plat_ – PEEP _tot_) from the analysis of ventilation tracings during occlusions; Vt, tidal volume; P _plat_, plateau pressure; PEEP _tot_, total positive end-expiratory pressure.

Table S2 Cardiopulmonary characteristics of the patients

| Patient NO. | RV function | Presence of pulmonary hypertension | Acute cor pulmonale | NSTEMI | Number of chest X-ray quadrants infiltrates(no.) | ILD | IPF | Other chronic obstructive airway condition | Acute or chronic PE | CTEPH | Pulmonary a-v physiology |
| --- | --- | --- | --- | --- | --- | --- | --- | --- | --- | --- | --- |
| 1 | Normal | No | No | No | 1 | No | No | No | No | No | No |
| 2 | Normal | Mild | No | No | 2 | No | No | No | No | No | No |
| 3 | Normal | No | No | No | 4 | No | No | No | No | No | No |
| 4 | Normal | No | No | No | 2 | No | No | No | No | No | No |
| 5 | Normal | No | No | No | 2 | No | No | No | No | No | No |
| 6 | Normal | No | No | No | 2 | No | No | No | No | No | No |
| 7 | Normal | Mild | No | No | 3 | No | No | No | Yes | Yes | No |
| 8 | Normal | No | No | No | 1 | No | No | Asthma | No | No | No |
| 9 | Normal | No | No | No | 1 | No | No | No | No | No | No |
| 10 | Normal | Moderate | No | No | 2 | No | No | No | Yes | Yes | No |

RV, right ventricular; NSTEMI, Non-ST Segment Elevation Myocardial Infarction; ILD, Interstitial Lung Disease; IPF, idiopathic pulmonary fibrosis; PE, pulmonary embolism; CTEPH, Chronic Thromboembolic Pulmonary Hypertension; a-v, arterio-venous.

# Additional Figures


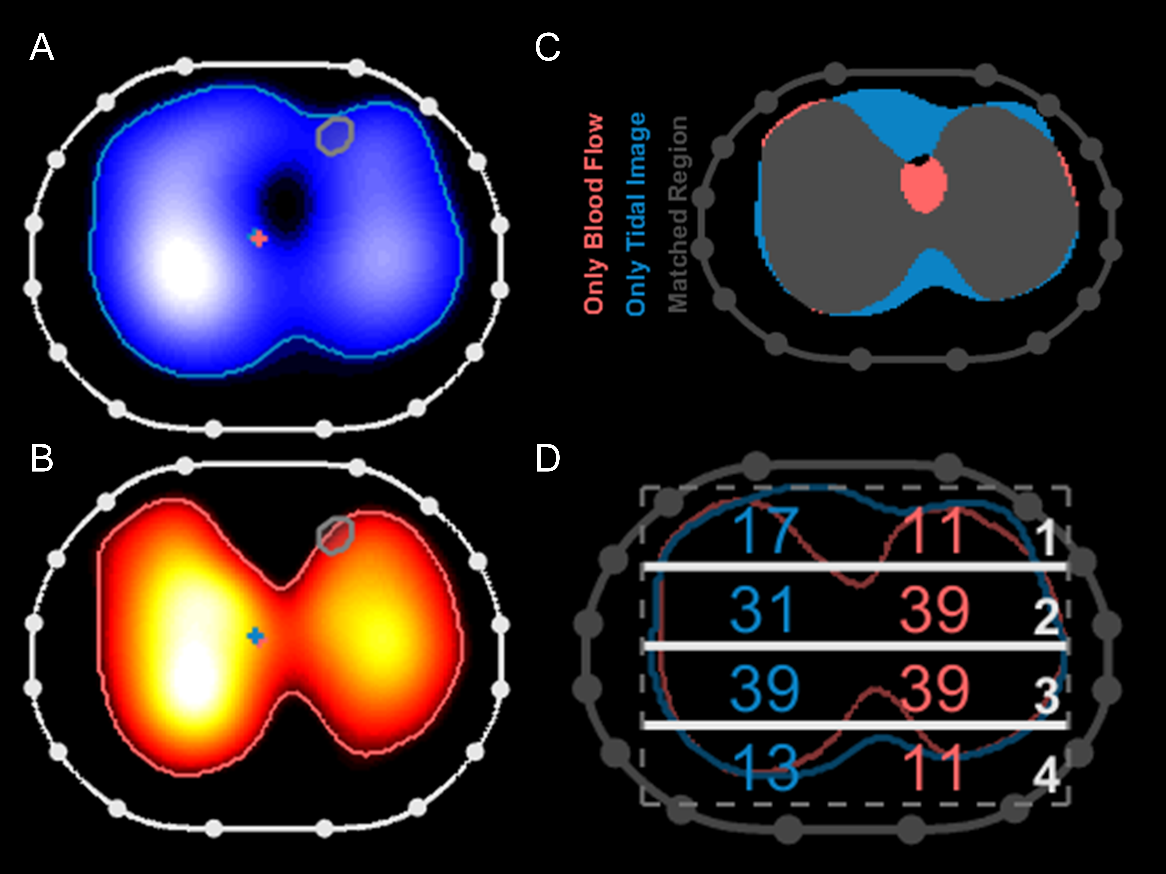


**Figure S1. Ventilation and perfusion measured by EIT in a representative patient.** A. Representative image of the ventilation (blue-color map) distribution. B. Representative image of the perfusion (red-color map) distribution. C. Representative map obtained by integrating ventilation and perfusion maps: the grey area indicates matched units which are both ventilated and perfused, while red area indicated only perfused units and blue area only ventilated units. D. Representative map with the percentage of ventilation (blue numbers) and perfusion (red numbers) distribution in the four horizontal regions of interest (ROIs). This choice allowed us to obtain more superimposable regions of interest.


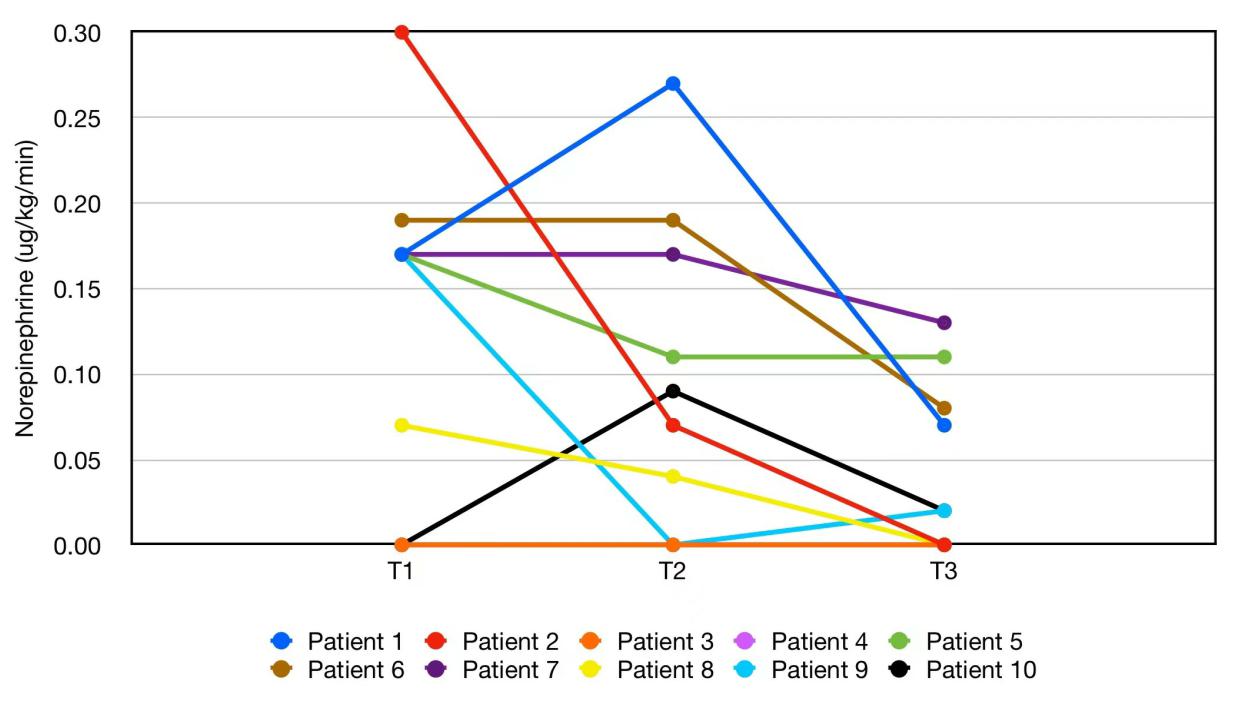


**Figure S2.** **The change in norepinephrine dose during the prone position for each patient.**


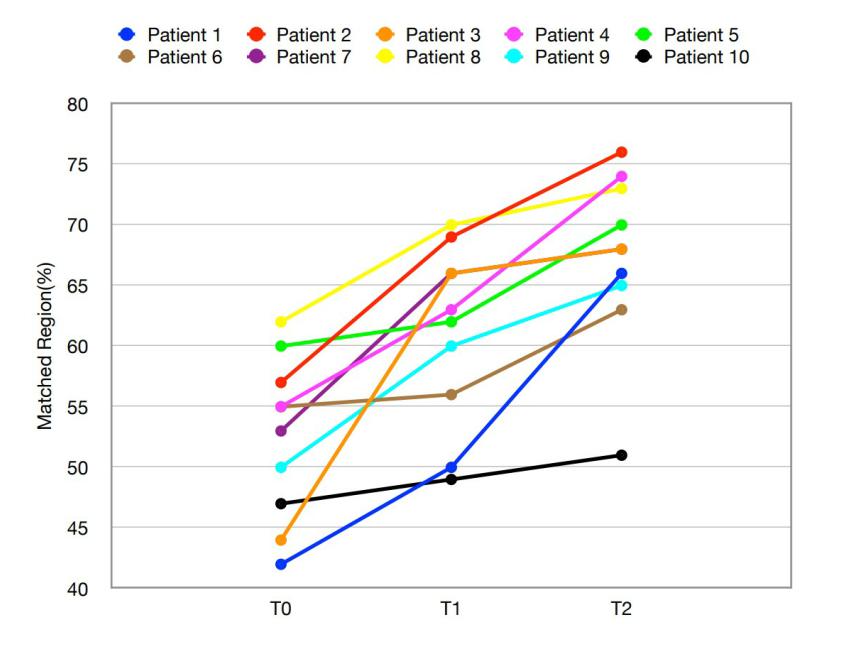


**Figure S3. A. The change in Matched Region (%) during the prone position for each patient.**


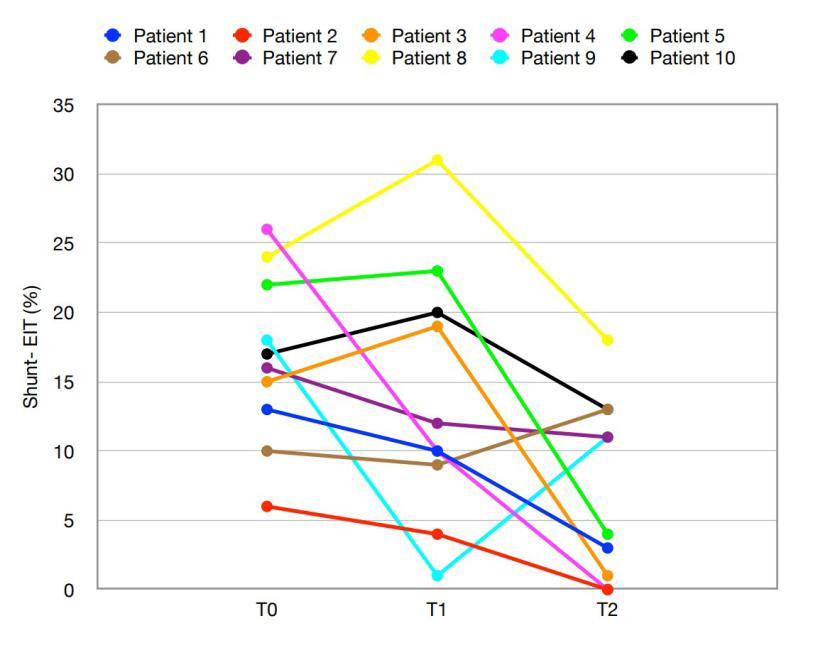


**Figure S3. B. The change in Shunt-EIT (%) during the prone position for each patient.**


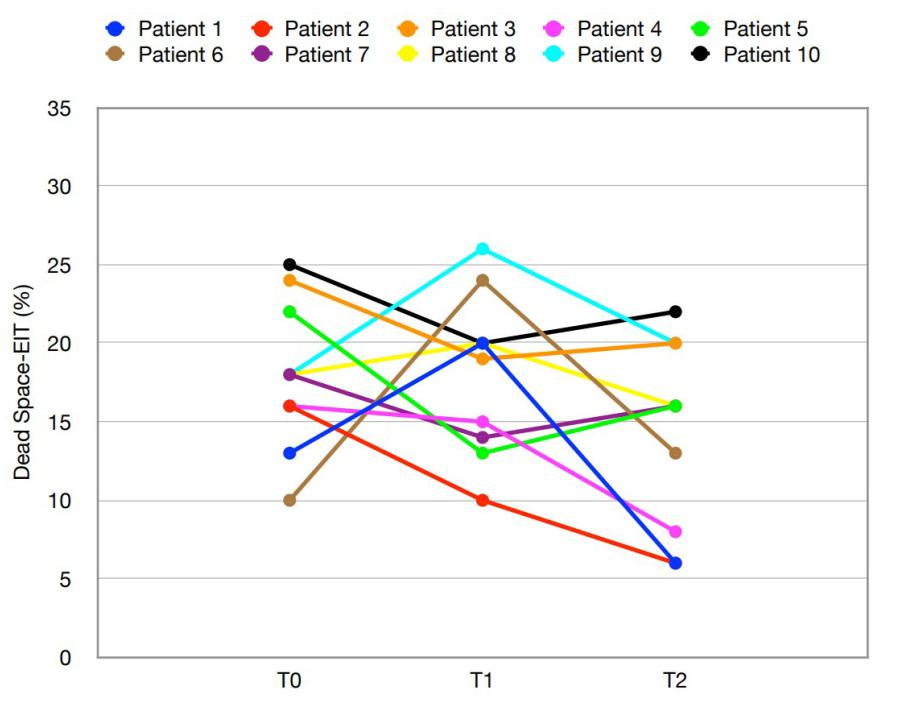


**Figure S3. C. The change in Dead Space (%) during the prone position for each patient.**


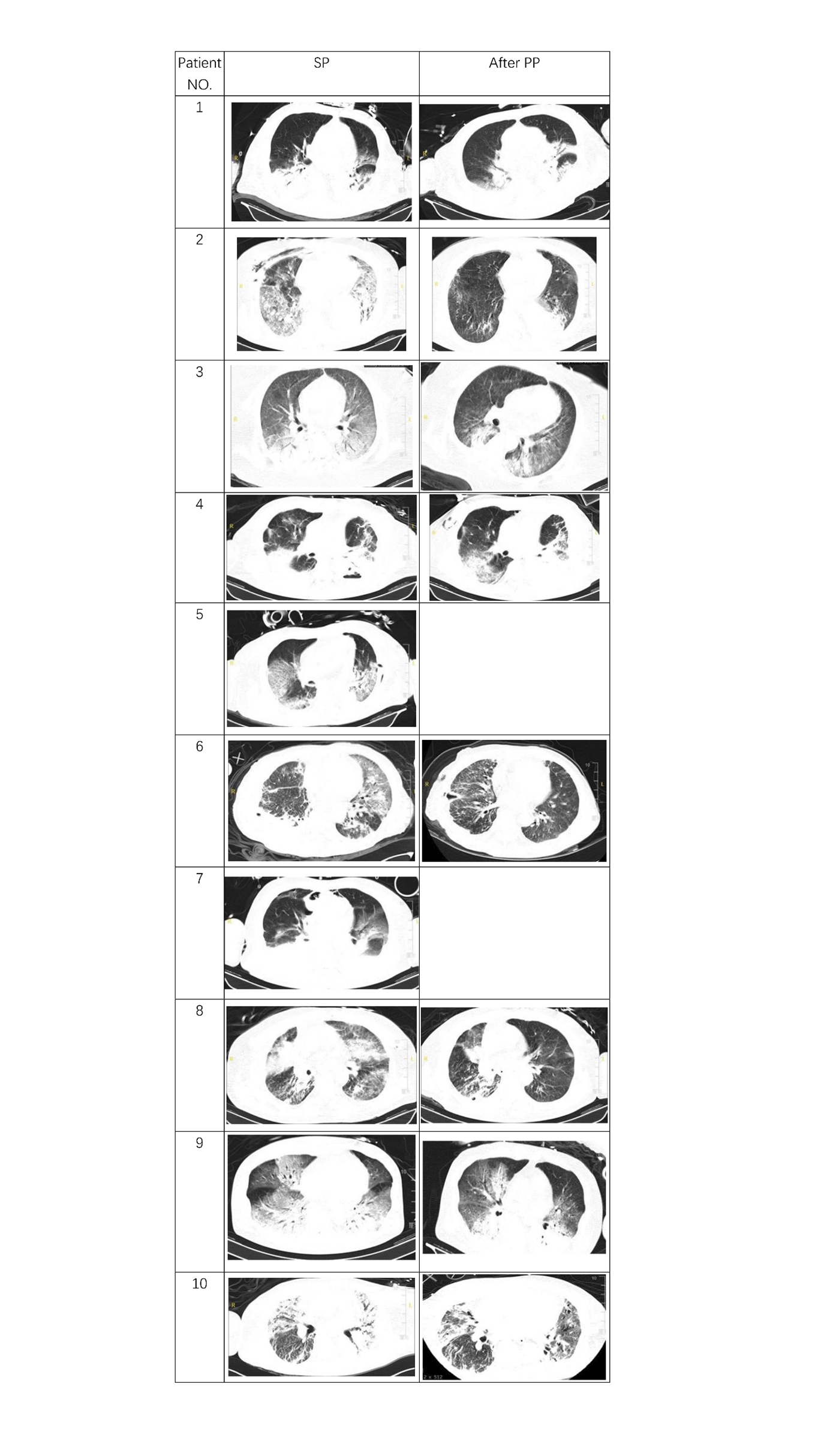


**Figure S4. Representative chest CT images obtained before prone position and after three prone position sessions.**


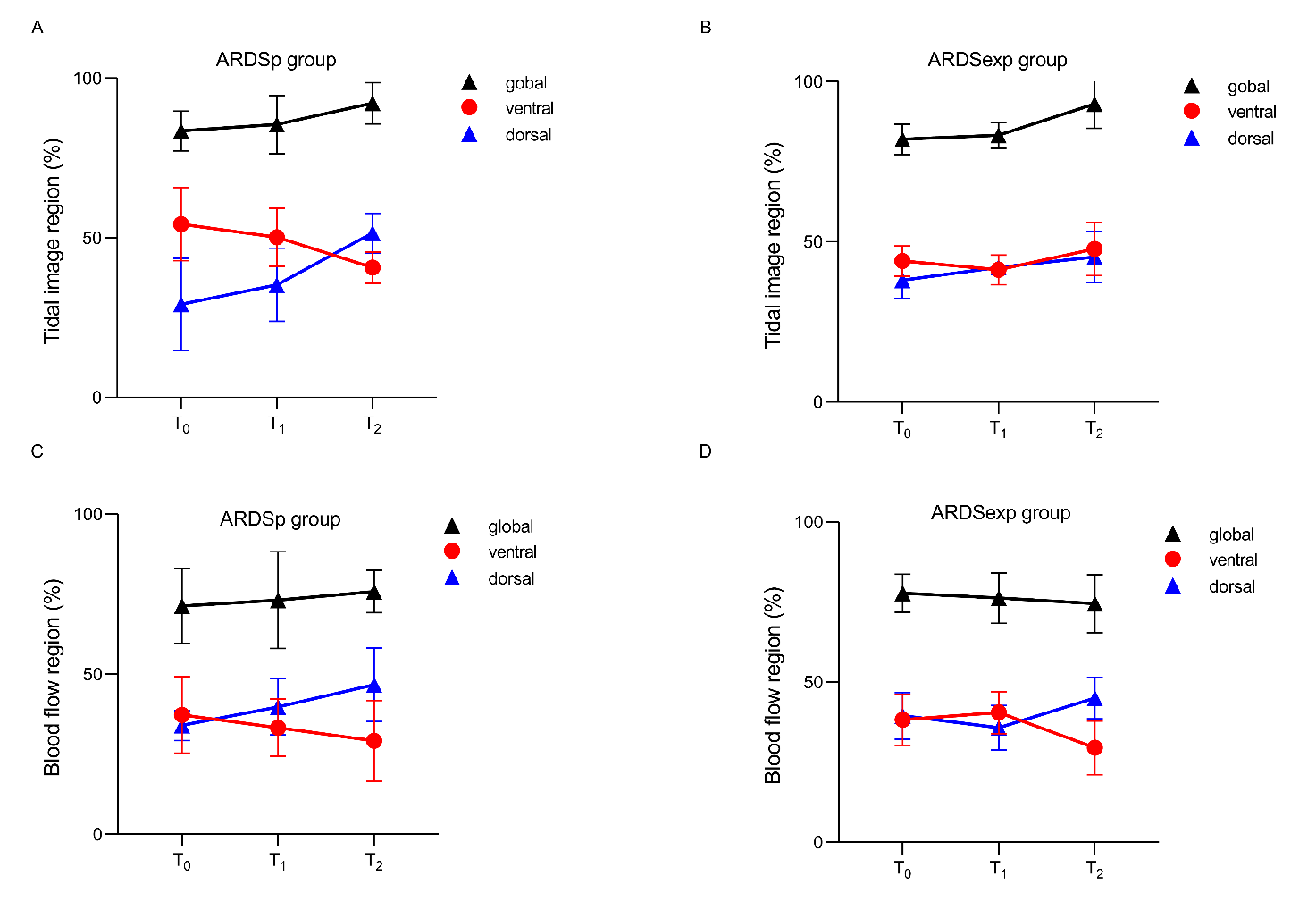


**Figure S5. Evolution of Tidal image region (%), Blood flow region (%) at T_0_, T_1_ and T_2_ in the ARDS _p_ and ARDS _exp_ groups.** Six patients could be categorized to the pulmonary cause (ARDS _p_) group, and four patients to the extrapulmonary cause (ARDS _exp_). In terms of the trend of change, the effect of prone position was more pronounced early in the ARDS _p_ group compared to the ARDS _exp_ group. However, prolonged prone ventilation finally both increases dorsal ventilation and perfusion in the lung in two groups, which results in improved ventilation-perfusion matching.
